# New Insights into *Listeria monocytogenes* Antimicrobial Resistance, Virulence Attributes and Their Prospective Correlation

**DOI:** 10.3390/antibiotics11101447

**Published:** 2022-10-21

**Authors:** Mahmoud E. Elsayed, Marwa I. Abd El-Hamid, Attia El-Gedawy, Mahmoud M. Bendary, Reham M. ELTarabili, Majid Alhomrani, Abdulhakeem S. Alamri, Saleh A. Alghamdi, Marwa Arnout, Dalal N. Binjawhar, Mohammad M. Al-Sanea, Amira I. Abousaty

**Affiliations:** 1 Department of Bacteriology, Immunology, and Mycology, Faculty of Veterinary Medicine, Suez Canal University, Ismailia 41522, Egypt; 2 Department of Microbiology, Faculty of Veterinary Medicine, Zagazig University, Zagazig 44511, Egypt; 3 Department of Bacteriology, Animal Health Research Institute, Dokki, Giza 12618, Egypt; 4 Department of Microbiology and Immunology, Faculty of Pharmacy, Port Said University, Port Said 42511, Egypt; 5 Department of Clinical Laboratories Sciences, The Faculty of Applied Medical Science, Taif University, Taif 26432, Saudi Arabia; 6 Centre of Biomedical Science Research (CBSR), Deanship of Scientific Research, Taif University, Taif 26432, Saudi Arabia; 7 Veterinary Quarantine, Cairo Airport, Zagazig 44511, Egypt; 8 Department of Chemistry, College of Science, Princess Nourah Bint Abdulrahman University, Riyadh 11671, Saudi Arabia; 9 Pharmaceutical Chemistry Department, College of Pharmacy, Jouf University, Sakaka 72341, Saudi Arabia; 10 Microbiology Department, Faculty of Science, Zagazig University, Zagazig 44511, Egypt

**Keywords:** listeriosis, foodborne, MDR, virulence, diversity

## Abstract

Listeriosis is one of the most common foodborne diseases caused by *Listeria monocytogenes* (*L. monocytogenes*). A poor prognosis has been recorded for the invasive listeriosis, especially neurolisteriosis. In several countries throughout the world, foodborne infections with *L. monocytogenes* exceeded the legal safety limits in animal sourced foods. Therefore, we decided to investigate the variability, virulence and antimicrobial resistance profiles of this pathogen. Both phenotypic and genotypic methods were used for identifying *L. monocytogenes* isolates and confirming their virulence profiles. The antimicrobial resistances and their correlation analysis with the existence of virulence genes were detected. Additionally, sequencing and phylogenetic analysis based on *L. monocytogenes inlA* and *inlB* genes were undertaken. The prevalence rate (11.9%) and the resistance profiles of *L. monocytogenes* were shocking. The multi-drug resistance (MDR) phenotypes were common among our isolates (64.9%). Fortunately, the resistance phenotypes were always associated with low virulence arrays and the MDR strains possessed low virulence fitness. Herein, the high genotypic and phenotypic diversity of *L. monocytogenes* isolates and their weak clonality and adaptability highlighted the difficulty in controlling and managing this pathogen. Therefore, it is important to add more restriction guidelines from national authorities on the consumption of ready to eat foods.

## 1. Introduction

*Listeria monocytogenes* is an animal and human pathogen, which is responsible for causing intracellular foodborne diseases as well as invasive listeriosis, encephalitis, endocarditis, perinatal infections, gastroenteritis, septicemia, meningitis, ophthalmitis and abortion. Life threatening listeriosis occurs among people with weakened immune systems, neonates, the elderly, and pregnant women [1]. The fatality rate among infected individuals is low; meanwhile, it may increase in the high-risk patients [2]. Regarding the animal infections, there are several forms of listeriosis, such as encephalitic and septicemic. The encephalitic form is characterized by neurologic signs such as depression and incoordination; meanwhile, the septicemic form may include depression, listlessness, emaciation and diarrhea [3].

In recent years, foodborne pathogens have caused major public health crises around the world. The incidence of foodborne diseases continues to grow, with high mortality and morbidity rates. These types of infections pose challenges in developing countries, especially in the Middle East and North Africa regions [4]. Several pathogens such as *Staphylococcus aureus*, *Salmonella* Enteritidis, enterohaemorrhagic *Escherichia coli* and *L. monocytogenes* can be transmitted via food chains [5,6,7,8].

*L. monocytogenes* can infect both humans and animals such as domestic pets, rodents, livestock, fish, avian species, and rabbits. The principle route of transmission of listeriosis is contaminated food, which has been estimated to be the source of infection in about 99% of the cases [9]. *Listeria monocytogenes* is commonly recovered from various types of poultry and meat products and it can grow well in dry sausage and in products with high pH values [10]. For that, public health officials should be watchful and should reassess manufacturing practices for processed poultry, rabbit and beef products and milk in order to decrease the spread of listeriosis.

Of note, *L. monocytogenes* had many virulence factors including listeriolysine O encoded by *hlyA* gene, internalins encoded by internalin (*inl*) genes, fibronectin-binding protein (FbpA), actA protein and enzymes such as lecithinase, serine protease and zinc metal protease. Additionally, *L. monocytogenes* is found everywhere in food processing and distribution, and it has contaminated a wide range of foods and animal products such as raw meat products, cheese, raw milk, and salads due to poor hygienic conditions [11]. The failure in management approaches with regard to this pathogen was attributed to its ability to form biofilms and its intrinsic physiological resistance against low temperature and high salt concentrations. Additionally, the antimicrobial resistance fitness of *L. monocytogenes* could be increased by the acquisition of resistance genes from *Enterococcus* and *Streptococcus* species [12,13]. Therefore, it is important to perform the in vitro evaluation of the antimicrobial agent before starting the treatment [14,15], and to find new and alternatives antimicrobial agents [16,17]. Delay in treatment in addition to the wide spreading of multidrug resistant (MDR) *L. monocytogenes*, which harbor many virulence factors, are considered the main causes for developing the infection [18]. In light of the above mentioned threats and high case fatality rates caused by *L. monocytogenes*, especially in Egypt, we found that it is timely and urgent to study the pathogenicity, virulence and antimicrobial resistance of this pathogen.

## 2. Results

### 2.1. Prevalence of L. monocytogenes from Different Sources and Governorates

**Figure 1:**
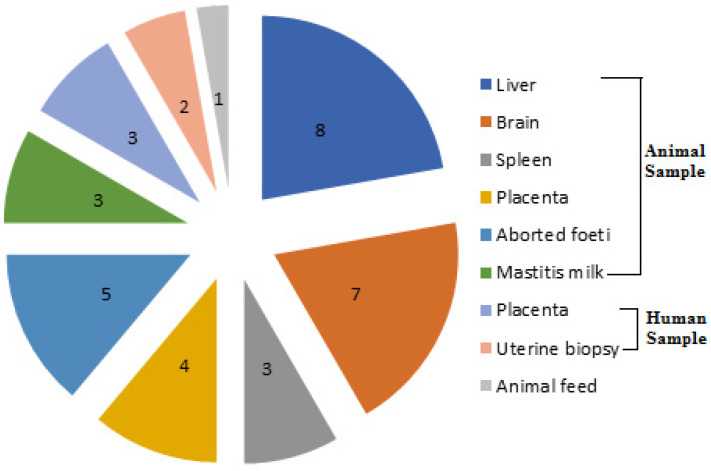
Number of *L. monocytogenes* isolates recovered from different sample types of animal, human and animal feed sources.

Out of the 310 samples screened in our study, 37 *L. monocytogenes* isolates were recovered with an overall prevalence rate of 11.9% comprising 13.5% (31/230), 10% (5/50) and 3.3% (1/30) in animal, human and animal feed sources, respectively. The recovered isolates were identified on the basis of their cultural characteristics, biochemical reactions, and via the Microbact™ Listeria 12L system. These isolates exhibited positive results for Anton’s eye, CAMP and hemolysis tests, and possessed the specific *16S rRNA* gene. Therefore, genotypic identification was in accordance with phenotypic characterization of *L. monocytogenes* isolates. The distribution of *L. monocytogenes* from different sources is illustrated in Figure 1. Regarding the animal origin, the majority of our isolates (26.7%, 8/30) were recovered from liver samples of sheep suffering from septicemia. Moreover, the high proportion of *L. monocytogenes* isolates from human sources was from placental samples (12%, 3/25), and only one isolate was obtained from 30 animal feed samples (3.3%). Notably, Sharkia had the highest prevalence rate of *L. monocytogenes* isolates (13.8%, 18/130), followed by Giza (12.2%, 11/90) and then Qaliubia (8.9%, 8/90) Governorates. 

### 2.2. Antibiogram Typing and Virulence Gene Profiles of L. monocytogenes

**Figure 2:**
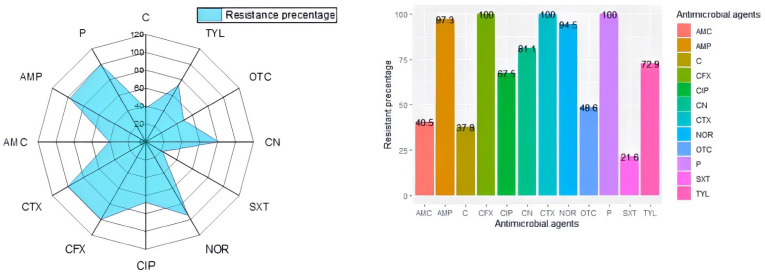
Resistance rates of *L. monocytogenes* isolates to the tested antimicrobial drugs. NOR: norfloxacin, CIP: ciprofloxacin, P: penicillin, AMP: ampicillin, CFX: cephalexin, CTX: cefotaxime, CN: gentamycin, C: chloramphenicol, TYL: tylosin, OTC: oxytetracycline and SXT: sulfamethoxazole-trimethoprim and AMC: amoxicillin-clavulanic acid.

According to the antimicrobial susceptibility results, *L. monocytogenes* isolates showed high resistance rates to penicillin, cephalexin and cefotaxime (100% each), followed by ampicillin (97.3%); meanwhile, the highest sensitivity levels were recorded to sulfamethoxazole-trimethoprim (78.4%) and amoxicillin-clavulanic acid (64.9%), as shown in Figure 2. Unfortunately, the majority of our isolates (24/37; 64.9%) showed the MDR pattern being resistant to three or more antimicrobial drugs from different classes. Moreover, MAR indices of the recovered isolates ranged from 0.17 to 0.42, indicating that they were originated from high-risk contamination. Regarding the distribution of the tested virulence genes among the recovered isolates, our results documented that all *L. monocytogenes* isolates possessed both *inlA* and *inlB* genes; meanwhile, 73% (27/37) and 64.9% (24/37) of the examined isolates harbored *prfA* and *hlyA* genes, respectively.

### 2.3. Phenotypic and Genetic Diversity of L. monocytogenes

**Figure 3:**
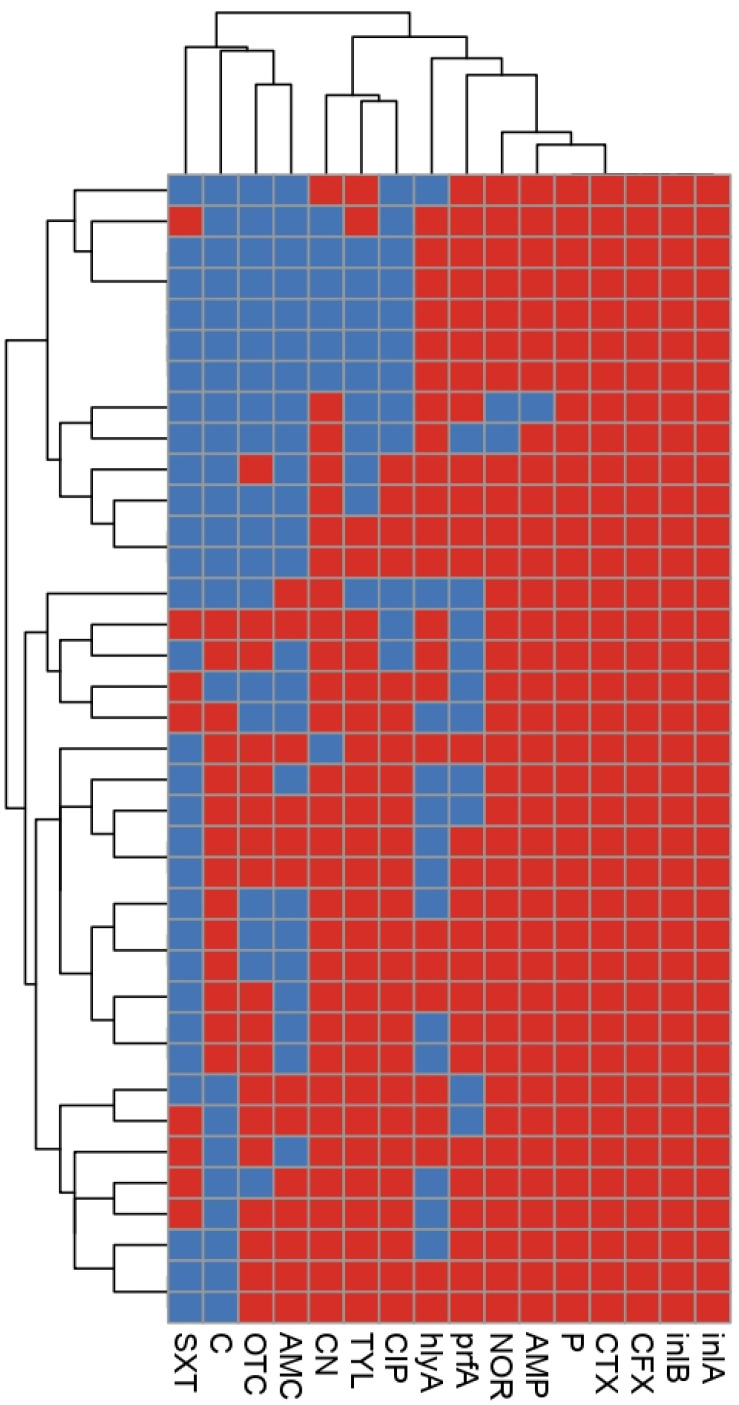
Heatmap showing the distribution of 37 *L. monocytogenes* isolates based on both virulence genes’ profiles and antimicrobial resistance patterns. The absence and presence of a particular virulence gene or sensitivity and resistance to a certain antimicrobial are indicated by blue and red colors, respectively. The text labels on the bottom specify the tested virulence genes and antimicrobial agents. Each row denotes one investigated isolate. The tree on the left displays the relatedness of the examined isolates according to their virulence genes’ profiles and the resistance they confer. AMP: Ampicillin, NOR: norfloxacin, P: penicillin, CFX: cephalexin, CTX: cefotaxime, CIP: ciprofloxacin, TYL: tylosin, CN: gentamycin, AMC: amoxicillin-clavulanic acid, OTC: oxytetracycline, C: chloramphenicol, SXT: sulfamethoxazole-trimethoprim, *inlA*: internalin A, *inlB*: internalin A, *prfA*: positive regulatory factor and *hlyA*: haemolysin.

Based on both antibiogram typing and virulence gene profiles, all of our recovered *L. monocytogenes* isolates were cloned into 28 clusters (Figure 3), which reflected the high degree of heterogeneity among these isolates. With the exception of six clusters, each isolate belonged to a unique lineage with a specific phenotypic and genotypic profile. The discriminatory power for combined phenotypic (antibiogram typing) and genotypic (virulence genes’ profiles) methods was 0.98, indicating the high variability of the tested isolates. Out of the six clusters, one contained five isolates; but 10 isolates belonged to the other five clusters (two isolates for each cluster) as shown in Figure 3.

### 2.4. Correlation Analysis

**Figure 4:**
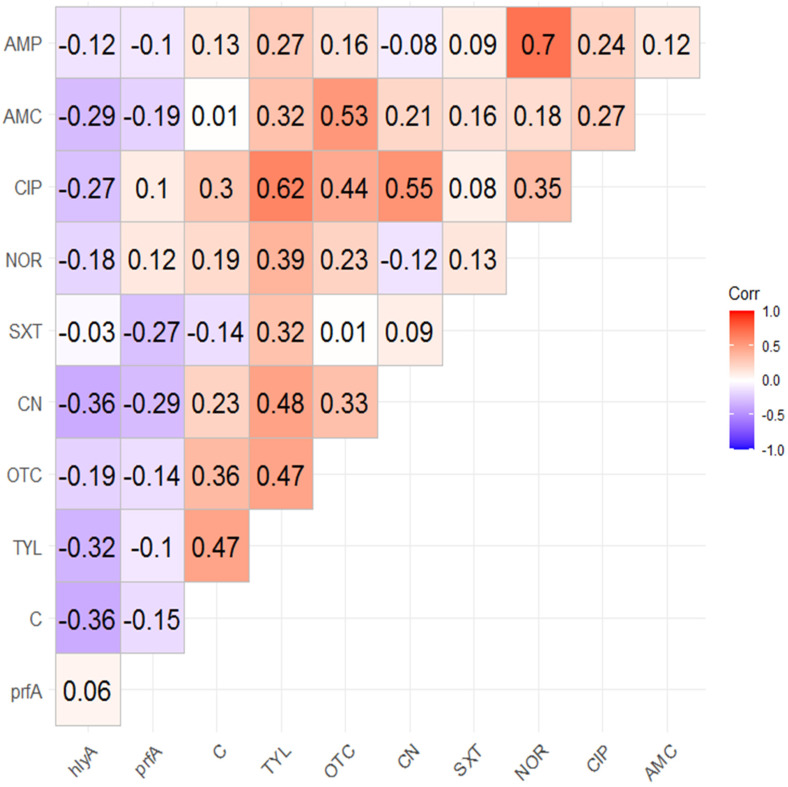
Heatmap illustrating the correlation coefficient (*r*) among and between resistances to the investigated antimicrobials and the presence of the tested virulence genes. The deep blue color reflects strong negative correlations; meanwhile, the deep red color indicates strong positive correlations. CIP: ciprofloxacin, TYL: tylosin, CN: gentamycin, C: chloramphenicol, AMC: amoxicillin-clavulanic acid, OTC: oxytetracycline, AMP: Ampicillin, NOR: norfloxacin, SXT: sulfamethoxazole-trimethoprim, *prfA*: positive regulatory factor and *hlyA*: haemolysin.

The correlation analysis among and between resistances to the investigated antimicrobials and the presence of tested virulence genes are illustrated in Figure 4. The results revealed that there were negative correlations between the existence of *prfA* or *hlyA* gene and the resistance to all tested antimicrobial drugs, with the exception of the very weak positive correlations between the presence of *prfA* gene and the resistances to ciprofloxacin and norfloxacin. Of note, strong positive correlations were detected between resistances to ampicillin and norfloxacin (*r*-value = 0.7) and resistances to ciprofloxacin and tylosin (*r*-value = 0.62). On the other hand, strong negative correlations between the existence of *hlyA* gene and the resistances to each of chloramphenicol and gentamycin (*r*-value = −0.36 each) is presented in Figure 4.

### 2.5. Phylogenetic Analyses of inlA and inlB Genes of L. monocytogenes

**Figure 5:**
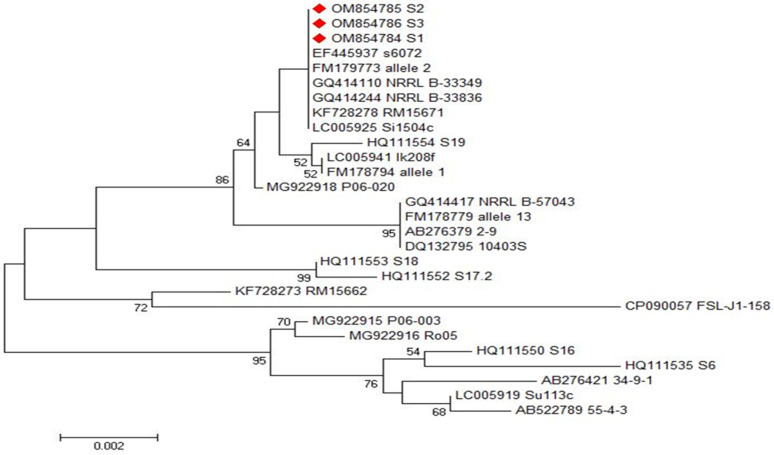
Phylogenetic analyses of *inlA* gene of the three investigated *L. monocytogenes* isolates (OM854784; S1, OM854785; S2 and OM854786; S3), illustrating the genetic relationships between the investigated isolates and those have been deposited in the GenBank database, as shown in the phylogenetic tree.

**Figure 6:**
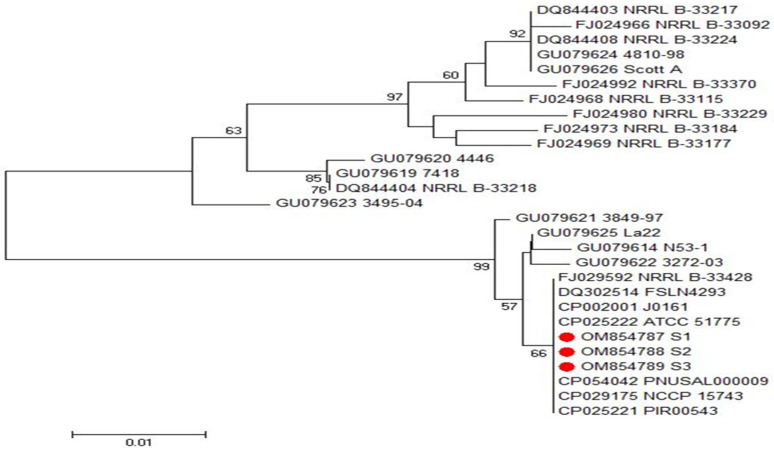
Phylogenetic analysis of *inlB* gene of the three investigated *L. monocytogenes* isolates (OM854787; S1, OM854788; S2 and OM854789; S3) illustrating the genetic relationships between the investigated isolates and those have been deposited in the GenBank database, as shown in the phylogenetic tree.

Phylogenetic and sequence analyses of *inlA* and *inlB* genes’ sequences among three representative *L. monocytogenes* isolates showing the highest resistance rates revealed that the examined isolates exhibited a remarkable genetic identity to other *L. monocytogenes* strains from different origins, which were deposited on the GenBank database, as demonstrated in Figure 5 and Figure 6. These sequences were deposited in the GenBank under the accession numbers of OM854784, OM854785 and OM854786 for *inlA* gene and OM854787, OM854788 and OM854789 for *inlB* gene. The final alignments of *inlA* gene consisted of 800 bp; 769 conserved and 31 variable sites; meanwhile, that of *inlB* gene comprised 343 bp; 304 conserved and 39 variable sites.

Based on the sequence analyses of both *inlA* and *inlB* genes, we did not find any new or unique sequences, and there were no new or unique mutations in these genes. Phylogenetic and sequence analyses of *inlA* genes revealed that our tested isolates (Accession No. OM854784, OM854785 and OM854786) displayed a complete identity (100%) to another food *L. monocytogenes* strain in Japan (Accession No. LC005925). Additionally, they showed high similarity percentages with *L. monocytogenes* food isolate in France (Accession No. FM178794, 99.9%), *L. monocytogenes* strain isolated from pig in Spain (Accession No. HQ111554, 99.7%), *L. monocytogenes* strain isolated from seafood in Japan (Accession No. AB276379, 99.5%), and *L. monocytogenes* strain isolated from watershed samples in the USA (Accession No. KF728273, 99.0%), as demonstrated in Figure 5. On the other hand, our *inlB* gene sequences (Accession No. OM854787, OM854788 and OM854789) displayed a remarkable genetic identity to other *L. monocytogenes* strains from different origins such as the food *L. monocytogenes* strain in South Korea (Accession No. CP029175, 100%), the *L. monocytogenes* strain isolated from cold smoked salmon in Denmark (Accession No. GU079625, 99.7%), the *L. monocytogenes* strain isolated from maternofetal interface in Denmark (Accession No. GU079621, 99.4%), the *L. monocytogenes* strain isolated from fish smoke house equipment in Denmark (Accession No. GU079614, 99.4%) and the *L. monocytogenes* strain isolated from spreadable sausage in Denmark (Accession No. GU079619, 93.6%), as demonstrated in Figure 6.

## 3. Discussion

Recently, infections from *L. monocytogenes* increased worldwide, and high death rates associated with this pathogen were recorded. Listeriosis is a serious disease that affects a wide range of animals and people, especially those with weakened immune systems, pregnant women and newborns. The prevalence rates of *L. monocytogenes* were variable among different sources in this study, with an overall percentage of 11.9%. The animal subjects were the most common source of *L. monocytogenes* isolates. This prevalence rate was higher than those recorded in previous studies conducted on different ecological niches in the same geographic area [19,20] reflecting the decreasing in awareness, carefulness and good practices in the Egyptian community. This pathogen can cause a zoonotic disease (listeriosis) in both humans and animals and it can be transmitted via several food chains such as meat, poultry and seafood products. Additionally, *L. monocytogenes* is widely spread and contaminates a wide range of foods and animal products such as raw milk, cheese, raw meat products and salads due to poor hygienic conditions [21]. *Listeria monocytogenes* can multiply in food to dangerous levels, even at refrigeration temperatures during distribution and storage [22]. Therefore, there is an urgent need to control *L. monocytogenes* at all stages in the food chains and manage its infection in animals. We can overcome this challenge by strengthening the good manufacturing and hygienic practices in all sectors of the food chain, avoiding the animal infections and rapid diagnosis and treatment of both animal and human infections.

Another crisis in *L. monocytogenes* infections is the wide spread of antimicrobial resistance. The MDR phenotypes are common among our isolates, which lead to treatment failure. The acquired resistance to antimicrobial drugs were rarely developed among *L. monocytogenes* isolates [23,24]; however, and in accordance with our findings, several recent reports have found increased rates of antimicrobial resistance among clinical food-borne pathogens [25,26,27]. Overall, *L. monocytogenes* isolates in this study exhibited MDR patterns to four classes of antibiotics: penicillins, quinolones, aminoglycoside, and macrolides. This is a great issue for public health, as it may pose a challenge in the treatment of listeriosis. Of note, *L. monocytogenes* exhibit varying degrees of resistances to the most common antibiotics. High levels of resistance to amoxicillin, cefotaxime, norfloxacin, tetracyclines and gentamicin were recorded in this study and in other studies [28,29]; meanwhile, the high susceptibility of our *L. monocytogenes* against trimethoprim/sulfamethoxazole was announced and is consistent with an earlier study [30]. The antimicrobial resistance in *L. monocytogenes* may be attributed to the acquisition of antibiotic resistance genes from other pathogens [27,31]. Moreover, the acquisition of conjugative transposons [32], active efflux associated genes [33], and ribosomal and chromosomal mutations [34] are the major mechanisms of *L. monocytogenes* resistance. Fortunately, sulfamethoxazole-trimethoprim and amoxicillin-clavulanic acid are still the alternative therapies in the case of unresponsiveness to the treatment with beta lactams’ antibiotics and intolerance to the first line therapies, as observed in our study and other reports [35,36].

Many factors affect the pathogenicity of *L. monocytogenes* involving numerous key virulence arrays such as internalins and haemolysin [37,38]. Notably, *L. monocytogenes* clinical isolates are multi-virulent; this was announced in several previous publications [39,40] as well as in our study. Previously, all clinical isolates possessed InlA and InlB proteins [41,42], which are essential for the recognition of several receptors and the invasion of different cell types. Furthermore, majority of our isolates showed hemolytic activities and harbored *hlyA* and *prfA* genes, which contribute to *L. monocytogenes* virulence and transmission. As expected from the correlation analysis, negative correlations between the existence of virulence genes (*hlyA* and *prfA*) and the antimicrobial resistances were detected.

Of note, the antimicrobial resistance genes were distributed between plasmid and chromosome [43]; however, the resistance genes can be acquired from the environment through plasmids and transposons [34]. Antimicrobial resistance, which enhances bacterial performance in vivo, may carry fitness costs, prompting a renewed interest in understanding the complex relationships between resistance and virulence. When both virulence and antimicrobial resistance genes are on the same mobile genetic element, a direct link can be observed [44]. The prediction of these correlations is highly challenging, as the bacterial genome is context-dependent and highly dynamic. Therefore, the correlation studies between the resistance to antimicrobials with each other, the existence of virulence genes with each other, in addition to the association between resistances to antimicrobials and the existence of virulence genes are very necessary owing to the continuous evolution in the genetic background. The costs of acquisition of antimicrobial resistance genes (increasing the resistance abilities) can be outweighed by benefits such as loss of virulence genes (decreasing in the virulence fitness) [14]. Fortunately, the high virulence arrays were associated with the sensitive phenotypes, and the MDR strains possess low virulence fitness [45]. This finding is accepted depending on the fixed genetic capacity of the bacterial genome. Moreover, the acquisition of resistance genes occurs in parallel with the loss of other genes such as virulence ones [46,47]. 

In this study, all *inlA* and *inlB* genes’ sequences were identical to the previous sequence deposited in the database of GeneBank considering different sources and geographic areas. We did not observe any new mutations among our genes sequences. This observation confirmed the slow evolution in the virulence genes compared to antimicrobial resistance profiles. Notably, most evolution in the virulence genes may lead to negative selection [48].

The control of listeriosis becomes more tedious due to the high degree of heterogeneity among *L. monocytogenes* isolates. Our report recorded the high variability level and the low host specificity among the tested *L. monocytogenes* isolates. In this context, several authors announced the weak clonality and adaptability of *L. monocytogenes* due to the high phenotypic and genotypic diversity, which is associated with different evolutionary processes within different hosts or in an ecological niche [49,50]. Therefore, specific control and prevention recommendations and more restricted isolation guidelines are urgently needed.

## 4. Materials and Methods

### 4.1. Ethics Statement

All study procedures were conducted in accordance with the guidelines of the Animal Ethics Review Committee of Suez Canal University (AERC-SCU201856), Egypt. Moreover, written informed consent was obtained from all patients.

### 4.2. Sampling

The study was conducted from April 2018 to November 2021. Three hundred and ten samples were collected from various sources at Sharkia (n = 130), Giza (n = 90) and Qaliubia (n = 90) Governorates, Egypt. The samples were collected from animal (n = 230), human (n = 50), and animal feed (n = 30) sources. The animal samples comprised the brain of sheep suffering from nervous manifestations (n = 30), placenta (n = 30), and aborted foeti (n= 30) of sheep suffering from abortion at the third trimester stage of pregnancy, lung (n= 30), liver (n = 30) and spleen (n = 30) of sheep suffering from septicemia, and finally mastitis milk (n = 50) of sheep suffering from mastitis. Human placental samples (n = 25) and uterine biopsies (n = 25) were collected from febrile and aborted women attending tropical hospitals. The samples were aseptically placed into sterile containers, kept in an ice box and transferred as soon as possible to the laboratory of the Bacteriology Unit at Animal Health Research Institute, Dokki, Giza for further bacteriological examination and the isolation of *L. monocytogenes*.

### 4.3. Isolation and Characterization of L. monocytogenes

*Listeria monocytogenes* isolates were phenotypically identified based on characteristic culture, morphological and biochemical features [51,52]. Furthermore, the recovered isolates were further identified using the Microbact™ Listeria 12L system (Oxoid, UK). The genotypic identification was used to confirm all *L. monocytogenes* isolates via a *16S rRNA* gene-based PCR assay [53].

### 4.4. Phenotypic Assessment of L. monocytogenes Virulence

#### 4.4.1. Anton’s Test

The test was performed by inserting 0.1 mL of *L. monocytogenes* suspension (10^9^ colony forming units) into the conjunctiva of one eye of rabbits, and the other eye was used as a control. Anton’s test positive results were recorded as purulent conjunctivitis within 24–48 h, followed by keratitis [54].

#### 4.4.2. CAMP (Christie–Atkins–Munch–Peterson) Test

The test was conducted, as described previously [55], using a standard beta-hemolytic *Staphylococcus aureus* (ATCC 25923) strain streaked in a straight line across the center of blood agar (Oxoid, UK) plates, followed by the streaking of identified *L. monocytogenes* strains in a direction perpendicular or vertical to the *S. aureus* culture without touching. The plates were subsequently incubated at 37 °C for 18–24 h and examined for the presence of hemolysis, which appeared as an arrowhead, circle, or rectangle in CAMP positive strains.

#### 4.4.3. Hemolytic Activity

Heavy pure colonies from tryptic soy broth yeast extract agar (Oxoid, UK) plates were inoculated onto 5% sheep blood agar (Oxoid, UK) plates and incubated at 35 °C for 24–48 h to detect hemolysis [56,57].

### 4.5. Antibiogram Patterns of L. monocytogenes Isolates

The Kirby-Bauer disc diffusion method was used to determine the antimicrobial susceptibility profiles of the retrieved *L. monocytogenes* isolates on Mueller-Hinton agar (Oxoid, UK) supplemented with 5% fresh defibrinated sheep blood using twelve different antimicrobial disks (Oxoid, UK): penicillin (P), ampicillin (AMP), amoxicillin-clavulanic acid (AMC), cefotaxime (CTX), cephalexin (CFX), ciprofloxacin (CIP), norfloxacin (NOR), sulfamethoxazole-trimethoprim (SXT), gentamycin (CN), oxytetracycline (OTC), tylosin (TYL) and chloramphenicol (C). Inhibition zones were measured and interpreted adopting EUCAST guidelines for *L. monocytogenes* [58]. To ensure the accuracy of the disc diffusion method, the MIC values for the investigated antimicrobials were detected by broth microdilution method according to EUCAST guidelines. Briefly, selected *L. monocytogenes* strains were streaked onto brain heart infusion agar plates and the plates were incubated at 37 °C for 24 h. Three to five colonies were picked and incubated in brain heart infusion broth for 6 h, and this culture was adjusted to 5 × 10^6^ CFU /mL using 0.9% NaCl solution. Ten microliters of this solution were used to inoculate 96-well microtiter plates containing Mueller-Hinton broth with different antibiotic concentrations. The *L. monocytogenes* strain LMEGY1 was utilized as a quality control organism. The isolates exhibiting resistance to at least one agent in three or more antimicrobial classes were categorized as MDR [59]. Multiple antibiotic resistance (MAR) indices were then calculated following the previously standardized formula [60]. The chance of a high risk source of contamination is always observed with high MAR indices. 

### 4.6. Molecular Detection of L. monocytogenes Virulence Genes

The PCR techniques were used to determine some virulence genes: *prfA*, *inlA*, *inlB* and *hlyA* in the recovered *L. monocytogenes* isolates [61,62,63]. The primers’ pairs used in PCR protocols and their predicted amplicons’ sizes are listed in Table 1. For genomic DNA extraction, the QIAamp DNA Mini Kit (Qiagen, Germantown, MD, USA, catalog no. 51326) was used. All PCR assays were carried out in triplicates and included positive control strains provided by the Animal Health Research Institute, Dokki, Giza, Egypt, with a DNA-free reaction as a negative control. The amplified PCR products were separated on electrophoresis gel (1.5% agarose stained with 0.5 μg/mL of ethidium bromide) and photographed.

### 4.7. Sequencing and Phylogenetic Analysis of L. monocytogenes inlA and inlB Genes

Direct sequencing of *inlA* and *inlB* genes among MDR *L. monocytogenes* isolates were carried out in both directions following purification with the QIAquick PCR Purification Kit (Qiagen, Germantown, MD, USA) using a Bigdye™ Terminator V3.1 cycle sequencing kit (Thermo Fisher Scientific, Waltham, MA, USA). The sequence similarity to GenBank accessions was determined using a Basic Local Alignment Search Tool. Phylogenetic trees were then created by the MegAlign module of Laser gene DNASTAR version 12.1 utilizing the neighbor-joining method in MEGA6.

### 4.8. Statistical Analysis

Hierarchical clustering and the correlation coefficient (*r*-value) between resistances to various antimicrobials in addition to the existence of virulence genes were evaluated using the R package corrplot, heatmaply, and GraphPad Prism (version 6; GraphPad Software Inc.; San Diego, CA, USA). Moreover, the discriminatory power of the typing methods used in the current study was assessed using Simpson’s index of diversity [64].

## 5. Conclusions 

Several ready to eat foods, especially those of an animal source, are always contaminated with *L. monocytogenes*, which is associated with serious public health implications worldwide. As observed among our tested *L. monocytogenes* isolates, this problem is compounded by the high prevalence rate, the wide spread of antimicrobial resistance, the heterogeneity and diversity of this pathogen. In contrast to the evolution in the virulence fitness, we observed acceleration in the acquisition of antimicrobial resistance among *L. monocytogenes* isolates, which increase the possibility of treatment failure with the available drugs. Therefore, we are in urgent need for new and alternative therapies. Our study spotlighted and predicted the serious health problems in the near future due to the evolution in *L. monocytogenes* strains. Therefore, we encourage the health organizations in all countries to take more stringent actions to hinder the spread of this resistant pathogen.

## Figures and Tables

**Table 1:** Target virulence genes, oligonucleotide primer sequences and fragments’ sizes used in PCR protocols for *L. monocytogenes*.

Target Gene	Oligonucleotide Primer Sequence (5′-3′)	Amplified Product (bp)	Reference
*16S rRNA*	F: GGACCGGGGCTAATACCGAATGAT AA R: TTC ATGTAGGCGAGTTGCAGC CTA	1200	[53]
*prfA*	F: TCTCCGAGCAACCTCGGAACC R: TGGATTGACAAAATGGAACA	1052	[61]
*inlA*	F: ACG AGT AAC GGG ACA AAT GC R: CCC GAC AGT GGT GCT AGA TT	800	[62]
*inlB*	F: CTGGAAAGTTTGTATTTGGGAAA R: TTTCATAATCGCCATCATCACT	343	[62]
*hlyA*	F: GCATCTGCATTCAATAAAGA R: TGTCACTGCATCTCCGTGGT	174	[63]

## Data Availability

All generated data in this report are available in the submitted manuscript.
